# Gender Differences in Atherosclerotic Vascular Disease: From Lipids to Clinical Outcomes

**DOI:** 10.3389/fcvm.2021.707889

**Published:** 2021-06-28

**Authors:** Tamar Vakhtangadze, Rajeeka Singh Tak, Utkarsh Singh, Mirza S. Baig, Evgeny Bezsonov

**Affiliations:** ^1^Department of Internal Medicine, New Vision University, Tbilisi, Georgia; ^2^Department of Biosciences and Biomedical Engineering, Indian Institute of Technology Indore, Indore, India; ^3^Laboratory of Angiopathology, Institute of General Pathology and Pathophysiology, Moscow, Russia; ^4^Department of Biology and General Genetics, I.M. Sechenov First Moscow State Medical University (Sechenov University), Moscow, Russia

**Keywords:** atherosclerosis, cardiovascular disease, ischemic heart disease, ischemic stroke, gender differences

## Abstract

Cardiovascular diseases (CVDs) are one of the main reasons of death and morbidity in the world. Both women and men have high rates of cardiovascular morbidity and mortality, although gender-related differences in mortality and morbidity rates are observed in different age groups of the population. In the large cohort of cardiovascular disease, ischemic heart disease (IHD), heart failure (HF), systemic hypertension, and valvular heart disease are particularly common in the population. CVDs caused by atherosclerosis are in the first place in terms of frequency, that is why society is particularly interested in this problem. The development and course of atherosclerotic processes associated with lipid and other metabolic changes are characterized by a long latent period, the clinical manifestation is often an acute vascular catastrophe, which can lead to human disability and death. Differences associated with sex are observed in the clinical course and manifestations, which raises the suspicion that gender influences processes related to atherosclerosis. Atherosclerotic cardiovascular disease (ACD) includes two main most dangerous clinical manifestations: IHD and cerebrovascular disease (mainly ischemic stroke). Other less common clinical manifestations of atherosclerosis include aortic atherosclerosis and peripheral vascular disease. Gender-related differences were also identified concerning these diseases. The present review discusses the effects of gender and age on atherosclerotic processes, disease development, and clinical manifestations. The metabolic basis for the development of atherosclerosis appears to be related to sex hormones. Thus this issue is interesting and useful for doctors of different specialties.

## Introduction: Prevalence of Atherosclerotic Cardiovascular Diseases in the Population

Ischemic heart disease (IHD) and stroke are the most common causes of death in the population. Barquera et al. analyzed data from the Global Burden of Disease Study and found that the death rate per 100,000 population in 2013 was 247.9 deaths, with 84.5% of deaths associated with CVDs and 28.2% of all-cause mortality (1). The same study reveals a declining trend in the share of cardiovascular disease worldwide, which equally reflects the morbidity and mortality rates of women and men, although there is an increase in absolute morbidity figures, an increase in the age of the patient, an increase in chronic forms.

IHD in women usually develops after an average of 7–10 years compared to men. According to the latest data, the prevalence of ischemic heart disease has increased significantly in young women due to unfavorable lifestyle changes over the last decade (1–3).

IHD is a major factor contributing to morbidity and mortality of women. Traditional Framingham risk factors such as high blood pressure, hyperlipidemia, diabetes, tobacco use, and unfavorable lifestyle habits such as unhealthy diet and a sedentary lifestyle contribute to the development of IHD (2).

Advances in diagnosis and treatment of cardiovascular diseases have led to declining morbidity and mortality rates, especially in developed countries, however, this reduction is not similar for men and women. Thus, between 1980 and 2002, overall the age-adjusted mortality rate decreased by 52% in men and 49% in women. The average annual decline rate for men was 2.9% in the 1980s, rising to 2.6% in the 1990s, and the average annual decline for women was 2.6%, which dropped to 2.4%. From 2000 to 2002, the average annual decline was the same−4.4% for both men and women (3).

There are some differences in the clinical course of diseases. Men are three times more likely than women to develop acute coronary syndromes (ACS), ST-elevation myocardial infarction (STEMI), or non-ST elevation myocardial infarction in the population under the age of 60, although this tendency decreases with age and over 75 years statistical changes are observed—the morbidity rate in women increases significantly, which is especially clear in relation to stroke (4).

Statistics on stroke deserve special attention and one of the causes of stroke is atherosclerotic vascular disease. Epidemiological studies of high reliability have established that women have a higher risk of developing stroke than men. Based on an analysis of US stroke study statistics, Rebecca W. Perskey and co-authors conclude that:

- Every fifth woman has a stroke
- More than 55,000 women have a stroke each year
- Stroke is the 4th leading cause of death in women
- Stroke kills 80,000 women a year
- The highest prevalence of stroke among women is in black women.

Not only is atherosclerosis linked to stroke, but women also have some gender-related conditions which may increase the risk of development of stroke, however, some of them have shown the association with atherosclerotic vascular disease as well. The high risk of stroke in women can be due to pregnancy—especially in the third trimester and postpartum period, preeclampsia—it is the high blood pressure that develops during pregnancy. Preeclampsia doubles the risk of stroke in a lifetime. Birth control pills can double the risk of stroke, especially in women with hypertension. Hormone replacement therapy does not reduce the chances of stroke. Migraine aura is associated with ischemic stroke in young women, especially if they smoke or use birth control pills. Atrial fibrillation increases the risk of stroke by 20% in women over 75 years of age (5).

## Common Risk Factors

**Figure 1:**
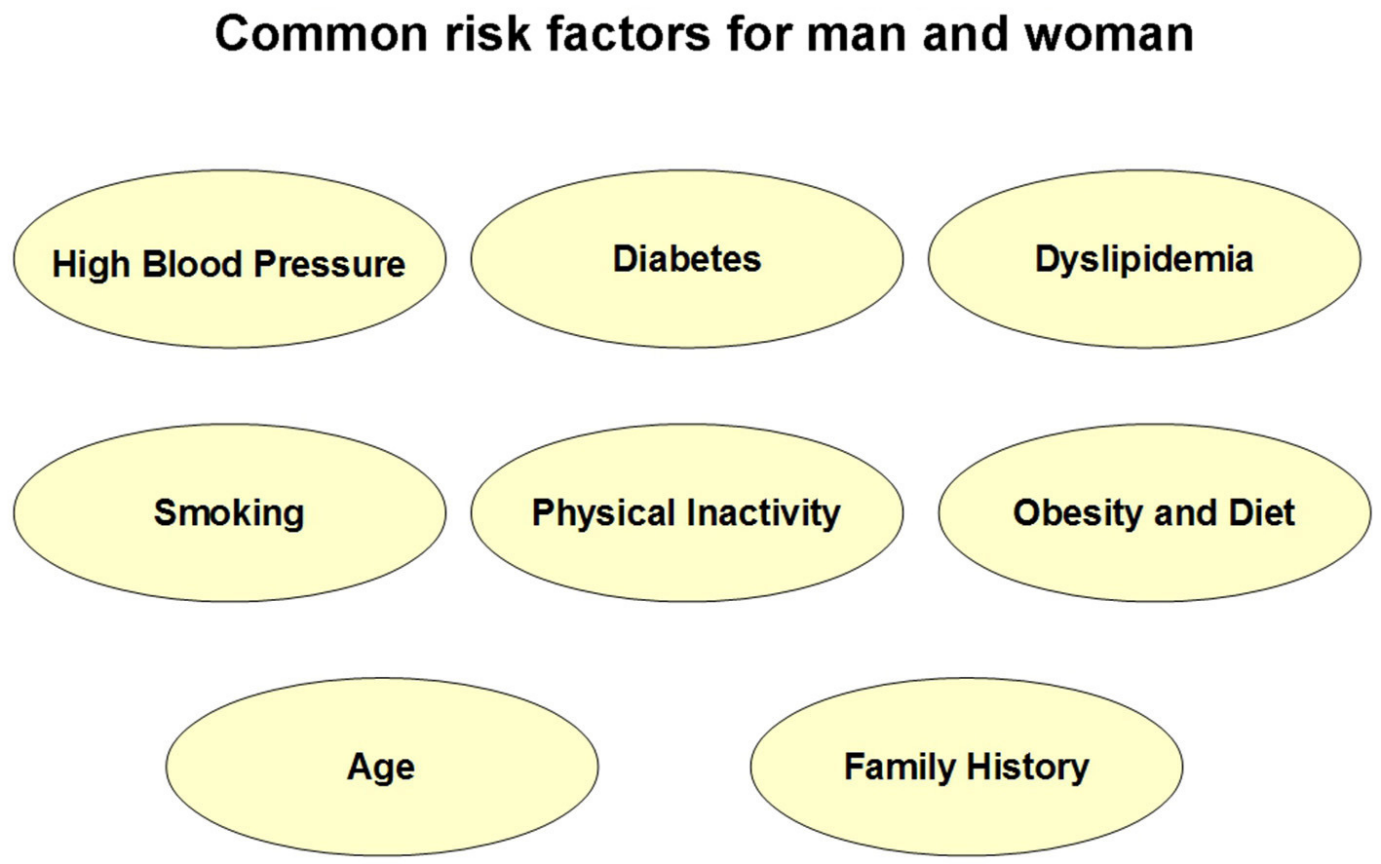
Common risk factors for man and woman.

Common risk factors—high blood pressure, diabetes, dyslipidemia, smoking, physical inactivity, obesity and diet, age, and family history are equally important for women and men (shown in Figure 1), although there are some differences associated with sex.

Smoking is a significant risk factor for atherosclerosis. Smoking women are more likely to have myocardial infarction than smoking men (6). Women who died of plaque rupture had a higher TG, HDL level did not show the difference from controls, while smoking was only a risk factor for plaque erosion (7, 8).

Smoking remains a significant problem for women−12.2% (95% CI, 11.4%−13.0%) of females in the USA reported cigarette use (compared to 15.8% of males), 7.2% of females smoked cigarettes during pregnancy. Distribution of other risk factors has shown differences also: the number of women reporting insufficient physical activity is 8% higher than in men; the prevalence of extreme obesity is significantly higher in females than in males−9.8% compared to 5.5%. High blood pressure and diabetes remain a major risk factor for ASCVD (9).

Numerous studies have shown that the presence of unique sex-related diseases such as PCOS (Polycystic Ovary Syndrome), Preeclampsia, Gestational Diabetes, and Menopause is important for women to increase the probability of developing atherosclerotic cardiovascular disease. It has been found that Diabetes Mellitus is also a very important risk factor for women.

CAD is uncommon in premenopausal women, especially when no other risk factors are present (10). The postmenopausal condition in terms of being a risk factor for the development of CAD in women is similar to that in men (11), and the incidence of MI in women significantly grows after menopause (10). Diabetes has also become evident as one of the major risk factors that deteriorate the ASCVD and CAD outcomes more in women than in men (12).

Cholesterol is one of the main causal risk factors for the development of atherosclerosis and CVD and is one of the seven indicators that AHA uses to determine CVH in children and adults. Among adults ≥20 years of age, the mean level of total cholesterol (TC) from 2015 to 2018 was 190.6 mg/dl. For men it was 187.7 mg/dl; for women it was 193.0 mg/dl (13, 14).

### Carotid Artery Intima-Media Thickening

Carotid Artery Intima-Media Thickening (CAIMT) is one of the well-established and important markers of atherosclerosis. The chronic inflammation of the arterial intima results from changes and interaction between the composition of LDL cholesterol, macrophages, T cells, monocytes, and smooth muscle cells of the arterial wall (15). The carotid intima-media wall thickness (CIMT) is a detection of the thickness of the tunica media and the tunica intima of the arteries, which is assessed by sonography of large arteries located close to the skin and the carotid artery. This measurement is used to detect a risk of developing atherosclerosis, to diagnose atherosclerotic vascular disease, and to track its regression or progression. CAIMT closely correlates with the prevalence of myocardial infarction or stroke (16). Assessment of CAIMT confirms the presence of atherosclerosis (Guideline atherosclerosis). Several studies have shown the association of CAIMT with gender-specific risk factors. CAIMT is one of the clearest diagnostic markers of atherosclerosis. It is known that CIMT is associated with visceral obesity, dyslipidemia, hyperinsulinemia, and increased systolic blood pressure, risk factors that also occur in PCOS (16).

Meyer et al. conducted a meta-analysis showing that CIMT is increased in women with PCOS compared to controls, indicating an increased risk of accelerated atherosclerosis in PCOS patients (17). Differences in TC levels in serum, as well as changes in body mass index, patience blood pressure, and the prevalence of diabetes helps to explain approximately 30% of the age-related increase in CHD (coronary heart disease) risk in men and 50–60% in women (14). Cholesterol is one of the main causal risk factors for the development of atherosclerosis and CVD and is one of the seven indicators that AHA uses to determine CVH in children and adults.

## Gender-specific Risk-Factors

**Figure 2:**
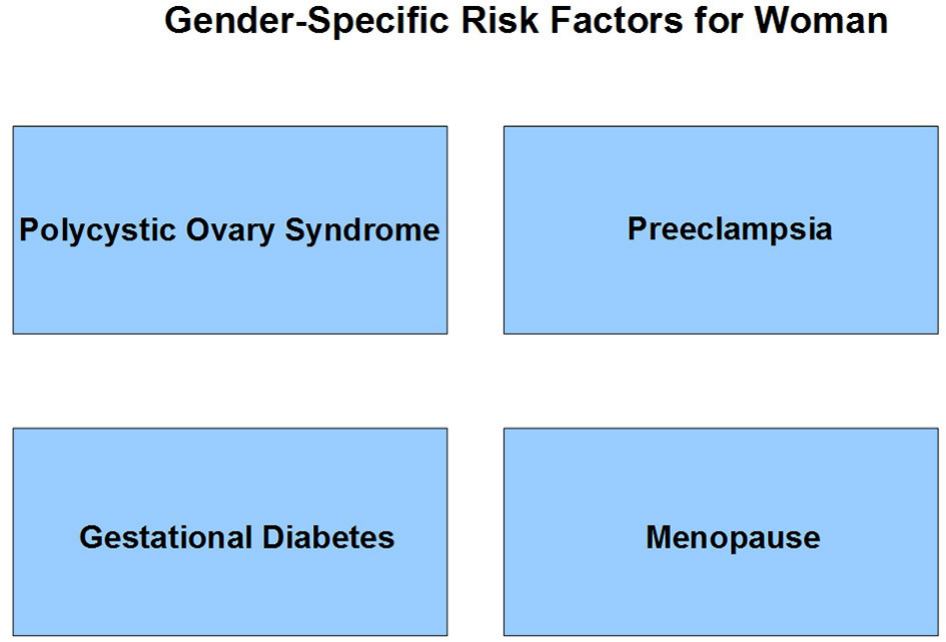
Risk factors specific for women.

Numerous studies have shown that women have unique gender-related diseases such as PCOS (Polycystic Ovary Syndrome), preeclampsia, gestational diabetes, and menopause (Figure 2). It has been found that diabetes is also a very important factor for women's health.

### Polycystic Ovary Syndrome

Polycystic ovary syndrome (PCOS) is an endocrinopathy happening with 6–10% of women of reproductive age and is manifested by hyperandrogenism, ovulatory dysfunction, and polycystic ovary syndrome with a complete phenotype. Talbott et al. studied the CAIMT condition in 125 women with PCOS under the age of 30 and concluded that the exposure during whole life to the cardiovascular risk profile in PCOS caused premature atherosclerosis and that the relationship between PCOS and intimate media thickness was partly because of weight and fat distribution and related risk factors. The results obtained suggest that (1) PCOS may lead to premature atherosclerosis in women with a lifelong negative cardiovascular risk profile, and (2) the PCOS-IMT connection is partly related to associated risk factors and weight and fat distribution. There may be an independent effect of PCOS that will not be explained by the above variables associated with this hormonal disorder.

### Preeclampsia/Eclampsia

Preeclampsia-Eclampsia is a violent gender-specific risk factor for women, which is a powerful complication of a young woman's pregnancy. However, it has been found that women who have contracted preeclampsia-eclampsia have an excessive risk of developing atherosclerotic diseases later in life.

Wu et al. conducted a systematic review and meta-analysis demonstrating the connection of preeclampsia with a 4-times higher incidence of heart failure and a 2-times higher risk of the development of CHD, stroke, and death because of CHD or CVD (18). Haukkamaa et al. performed a study that suggested that preeclampsia is an independent risk factor for the development of atherosclerotic plaque (19). Kessous et al. conducted a study concluding that previous pregnancy with preeclampsia serves as an independent risk factor for long-term maternal atherosclerotic morbidity. Patients with severe and recurrent episodes of preeclampsia have higher risk of atherosclerotic morbidity (20).

### Gestational Diabetes

These differences in outcomes are also observed in the case of young women suffering from gestational diabetes or type 1 diabetes (21, 22). Diabetes prevalence together with some other factors explains about a third of the age-related increase in CHD risk in men and up to 60% in women (14).

### Menopause

Estrogen signaling in liver can contribute to gender differences in atherosclerosis development by promoting the hepatic steps of reverse cholesterol transport (RCT) (23). The role of estrogen in the development of early steps of RCT can be controversial in humans. The cholesterol efflux ability of macrophages can be improved by estradiol-esters from HDL (24). The deficiency of estrogen in menopause increases the cholesterol efflux from HDL in comparison with premenopausal women, most probably because of increased VLDL-TG levels after menopause (25).

## Pathogenesis

The major pathogenetic mechanism of the development of vascular events is plaque erosion and rupture which leads to the development of thrombosis. Unstable angina, sudden death, and acute myocardial infarction can be combined in a group called acute coronary syndromes (ACS). Acute luminal thrombosis serves as the main cause of sudden coronary death in up to 65% of cases. The inclusionof total occlusion increases the incidence up to 75%, supporting the research made by Davies and Thomas (26). Plaque rupture, erosion, and calcified nodules are leading causes of thrombosis found during autopsy studies in sudden coronary death cases (27).

Acute thrombosis in men happened more often than in women (53 and 46%). Women with the age younger than 50 years old more often had plaque erosion in comparison with women older than 50 years old (84 and 32%). Plaque rupture was happened more often in women who were more than 50 years old in comparison to younger women (53 and 16%). The incidence of rupture was identical in all age groups (young and old) of men (75 and 69%), but younger men more often had cases of erosion than older men (29 and 18%). The cases of plaque erosion happened more often in women than men (58 and 24%) and rupture happened more often in men than women (71 and 33%).

The incidence of organized thrombus (CTO) was identical in men and women but happened more often in older than younger individuals of both genders and 84% of them had associated healed myocardial infarction. Clinical presentation of ischemia involves macrovascular and microvascular circulation. Nitric Oxide (NO) is a major determinant of microvascular flow. Vascular endothelium synthesizes NO, which is involved majorly in the processes like inhibiting platelet aggregation and adhesion and maintaining the basal vascular tone of arteries (28), inhibition of atherosclerotic plaque development (29), and vascular smooth muscle proliferation inhibition (30).

Various factors contribute to the development of atheroma formation, thrombosis, inflammation, endothelial nitric oxide synthase (eNOS), reactive oxygen species (ROS), matrix degradation being some of them (31).

The process of atherogenesis is composed of three major steps:

- Fatty streaks formation (these initial lesions of Atherosclerosis are caused by the focal increase in the lipoproteins of the intima layer of arteries);
- Atheroma formation;
- Atherosclerotic plaques formation.

The development of atherosclerosis is based on metabolic changes in the metabolism of lipids which is one of the main factors for this disease. There are major sex-based differences in cholesterol metabolism which most likely contribute to the large set of differences in rates of development and progression of atherosclerotic cardiovascular disease (32). The cholesterol-engorged macrophage “foam cell” is the hallmark and agent provocateur of atherosclerosis. The early lesions of atherosclerosis, so-called fatty streaks that can be detected in the first decade of life, are accumulations of foam cells in the arterial intima. In advanced fatty, fibrous, atherosclerotic plaques, foam-cell apoptosis produces a cholesterol-rich necrotic core, which makes plaques prone to rupture and thereby promotes vascular thrombosis. The liver X receptors (LXRs) α and β (LXRα and LXRβ) are master regulators of whole-body cholesterol homeostasis, intermediary metabolism and energy balance, and the integration of metabolic and inflammatory signaling (15).

It should be also mentioned that mitochondrial mutations are believed to play an important role in atherosclerosis pathogenesis but are left outside of the focus of this review.

Data collected from the study conducted to find the mechanisms providing gender differences in serum lipoprotein concentrations, the kinetic behavior of apoB-100 suggest that the mechanism for lower TRL-C (triglyceride c) and LDL-C (low-density lipoprotein c) concentrations in women was determined predominantly by higher TRL and LDL FCR (fractional catabolic rates) rather than lower PR (production rates). This study explains to a certain degree the lower CVD risk in premenopausal women in comparison with men (33).

There are differences in cholesterol profiles of men and women during aging. Low-density lipoprotein (LDL) levels are lower in women than in men until they reach age of 50, when LDL levels grow in women. High-density lipoproteins (HDL) are about 10 mg/dl higher in woman in comparison to men of all age groups. Lipoprotein (a) levels increase with age in perimenopausal women. The increase in CAD in older women can be explained by the above mentioned change in levels of lipids. It was confirmed after epidemiological studies that high cholesterol serves as a risk factor for CAD in women. A lower HDL value predicts the coronary risk in women better than in men (34).

Reviewing many studies, it can be concluded that estrogen replacement therapy may be useful to increase the release of NO in arterial vasculature in postmenopausal women. However, the data are controversial. Replacing estrogen has been shown to amplify hyperemia in the brachial artery by flow-mediated vasodilation (35), it has also shown an increase in acetylcholine mediated endothelial vasodilation by infusion of 17β-estradiol in the brachial artery (36). Thus, lack of estrogen seems to be involved in the pathogenesis of ischemia and atherosclerosis.

It is observed that pathophysiological mechanisms involved in the development of coronary artery atherosclerosis are sex–specific. There are also age-related differences in men and women in terms of disease progression (37). Men and women had almost identical mean age of the total population (women 50 ± 11, men 48 ± 10) which was lower than the one of the group with acute myocardial infarction (mean age 70 years) (38). Some important biological differences between men and women are related to differences in size of the arteries. Carotid arteries of women are smaller with less plaques but more obvious stenosis, which may be related to differences in remodeling. Women have lower risk of CVD in comparison with men even when levels of ovarian hormones decline with menopause (39, 40). CHD, coronary heart disease according to 2011–2014 NHANES data is around 20% in men and 11% in women in the range of 60 to 79 years of age, and similar trends for older individuals (41).

## Conclusions

Atherosclerosis is a major contributor to morbidity and mortality worldwide and this raises questions regarding the importance of research in this field. Sex-related issues of pathogenesis and clinical presentation of atherosclerosis are emerging direction for evaluation, determining similarities and differences and will influence prevention and treatment modalities. Exploring sex-based differences in atherosclerotic cardiovascular diseases is a need of the moment and the basis of differentiation could be related to the difference between major hormonal levels and genetic expression. The protective effects of the changes in the lipid profile were only 25% (41). Estrogen has different effects that can contribute to a cardioprotective effect as it plays a role in reducing the level of total cholesterol and low-density lipoprotein cholesterol, it also contributes to the elevation of high-density lipoprotein cholesterol, and helps in decreasing the levels of fibrinogen and factor VII (42). Similar outcomes can be observed in the case of lower risk of hypertension and lower lipid levels in premenopausal females compared to their male counterparts (43).

## Author Contributions

TV: conceptualization, supervision, and writing—original draft preparation. RS: writing—original draft preparation and bibliography. TV, RS, US, MB, and EB: writing—review and editing. All authors contributed to the article and approved the submitted version.

## Conflict of Interest

The authors declare that the research was conducted in the absence of any commercial or financial relationships that could be construed as a potential conflict of interest.
